# Genetic susceptibility to sarcoid in Arabian horses: associations with MHC class II and compound MHC class I/KLRA genotypes

**DOI:** 10.1007/s11259-025-10748-2

**Published:** 2025-05-01

**Authors:** Leona Vychodilova, Martin Plasil, Jan Futas, Andrea Kopecka, Dobromila Molinkova, Tamara Wijacki, Petr Jahn, Ales Knoll, Petr Horin

**Affiliations:** 1 Department of Animal Genetics, Faculty of Veterinary Medicine, University of Veterinary Sciences Brno, Brno, 61242 Czech Republic; 2 RG Animal Immunogenomics, CEITEC VETUNI, University of Veterinary Sciences Brno, Brno, Czech Republic; 3 Faculty of Agronomy, Mendel University, Brno, Czech Republic

**Keywords:** Horse, Sarcoid, MHC, KLRA, Association

## Abstract

Although the Major Histocompatibility Complex (MHC) has been repeatedly associated with susceptibility to equine sarcoid, a disease associated with bovine papillomavirus infection, the role of the MHC in the mechanisms of the disease is not fully understood. The objectives of our work were to analyze associations between polymorphic markers of the MHC genomic subregions and of the Natural Killer Complex (NKC) genomic region and the presence of sarcoid in Arabian horses. Microsatellite loci located in the MHC class I, II and III subregions and two MHC class II genes (*DRA*,* DQA1*), along with a set of NKC (KLRA, CLEC subregions) microsatelllite markers were genotyped. Fifteen microsatellites of the standard parentage kit, located outside the MHC and NKC regions, were tested as controls. Standard chi-square and Fisher tests with Bonferroni corrections were used for association analyses. Significant associations of MHC class II and MHC class I_KLRA polymorphic markers with the presence of clinical sarcoid were observed. These findings are consistent with biological theory and indicate a role of MHC class I, class II and KLRA molecules in adaptive as well as in innate immune responses to equine sarcoid. Although limited to Arabian horses, these data point to an as yet unadressed hypothesis regarding the possible roles of NK cells in the pathogenesis of equine sarcoid.

**Supplementary Information:**

The online version contains supplementary material available at 10.1007/s11259-025-10748-2.

## Introduction

Sarcoids are non-metastatic persistent fibroblastic neoplasms that can be locally invasive. Although they are one of the most common skin tumors found in horses, donkeys, and zebras (Knottenbelt 2005; Marti et al. 1993; van Dyk et al. 2009), their etiology is not fully understood. They occur in six different forms at specific predilection sites on the body (Ogłuszka et al. 2021). Due to their high tendency to recur, sarcoids are difficult to treat.

Bovine papilloma virus BPV1 and BPV2 play a role in the development of sarcoids (Chambers et al. 2003; Nasir and Campo 2008). The presence of viral DNA has been confirmed in most analyzed lesions (Martens et al. 2001). Contact with BPV may be an initiating factor, but it alone is insufficient for tumor proliferation; other factors such as skin damage, immune status, and genetic predisposition also contribute (Bogaert et al. 2005; Chambers et al. 2003). Both increased incidence in families (Ragland et al. 1970) and interbreed differences in the occurrence of sarcoids indicate a genetic basis for resistance/susceptibility to sarcoids. A higher prevalence of sarcoids has been found in Quarter Horses, Arabians, and Paint Horses, while they are less frequent in Thoroughbreds (Valentine 2006). Standardbred horses have a lower prevalence of sarcoids compared to Thoroughbreds (Angelos et al. 1988; Mohammed et al. 1992).

Mechanisms of immunity related to sarcoid development are not fully understood. As equine sarcoid fibroblasts exhibit features of cancer cells, they are believed to involve mechanisms associated with BPV infection and tumor fibroblast invasion. The presence of sarcoids in the horse’s body elicits differential expression of hundreds of genes, including immunity-related (IR) genes (reviewed by Ogłuszka et al. 2021). Genetic variation in immune responses against the virus and/or tumor cells is thus one of possible factors underlying the observed individual and interbreed differences (Staiger et al. 2016). Cytotoxic T lymphocytes and natural killer (NK) cells are two types of the mammalian immune cells capable of recognizing and killing the individual’s own cells (Vojdani et al. 2024).

The presentation of antigenic peptides by major histocompatibility complex (MHC) molecules is a crucial immune mechanism allowing recognition of virus-infected and/or tumor cells by T cells. MHC class I molecules present peptides derived from intracellular antigens, while MHC class II molecules are involved in the presentation of primarily extracellular antigens. Molecules encoded in the class III MHC subregion do not present antigens but are involved in several other immune mechanisms (Kulski et al. 2019). Due to the permanent selection pressure of everchanging pathogens, the MHC class I and II genes are the most polymorphic vertebrate genes. As such, they are prime candidates for disease association studies, including studies on equine sarcoids. At the population level, associations of sarcoids with both MHC class I and II candidate polymorphisms have been repeatedly reported (Broström 1995; Broström et al. 1988; Hörnaeus 2013; Lazary et al. 1994). With the advent of genome-wide association studies (GWAS), the MHC genomic region on ECA20, along with other chromosomal regions, was associated with sarcoid occurrence (Jandova et al. 2012; Staiger et al. 2016).

Transcriptome studies of BPV- 1-transformed equine fibroblasts and of sarcoid cells confirmed and extended this information to other IR genes, including innate immunity genes (Mählmann et al. 2014; Semik et al. 2017; Yuan et al. 2008) and miRNAs (Pawlina et al. 2017). In this context, strong downregulation of MHC class I molecules was observed in all tumors analyzed (Yuan et al. 2008). This finding was extended by Marchetti et al. (2009), who observed inhibition of MHC class I molecule expression by the BPV E5 oncoprotein.

These findings raise questions about the role of NK cells in the pathogenesis of equine sarcoids. Unlike cytotoxic T lymphocytes, NK cells can recognize cells lacking MHC class I molecules on their surface through specific inhibitory receptors which use MHC class I molecules as ligands. These receptors are activated when no MHC class I ligand is available on the cell surface, initiating a cascade which leads to target cell killing. The functional receptor-ligand relationships between MHC and NK cell receptors (NKR) molecules are reflected by associations between MHC/NKR composed genotypes and disease observed in human populations (Carrington et al. 2005; Orgul et al. 2021; Pollock et al. 2022; Rajagopalan and Long 2005). In horses, a family of expanded NK cell receptor *KLRA* (killer cell lectin-like receptor A) genes, originally named *Ly49*, encoding lectin-like receptors of NK cells has been identified (Takahashi et al. 2004). Genes of the KLRA family are part of a complex genomic region, the Natural Killer Complex (NKC), which also includes other genes, such as *CLEC* genes (Schwartz et al. 2017). Some KLRA genes are highly polymorphic in horse populations (Futas et al. 2020). Recently, we observed non-random associations between MHC and KLRA polymorphic markers in different horse populations (Bubenikova et al. 2024). However, there is no information on their association with equine sarcoid or with other equine diseases.

The objective of this study was to analyze associations between polymorphic markers of the major histocompatibility complex sub-regions and of the natural killer complex genomic region with the presence of clinical sarcoid in Arabian horses.

## Materials and methods

### Horses and diagnostics of sarcoid

The phenotype analyzed in this study was clinical manifestation (presence/absence) of sarcoid. A total of 43 Arabian-type horses originating from several (3) studs of the Czech Republic were studied. The sex and age of each horse was recorded at the time of examination. Sarcoids were diagnosed by detailed inspection and palpation of the entire body surface according to standard criteria described by Haspeslagh et al. (2018). Two healthy horses of the same breed and gender, of comparable or higher age, from the same environment in terms of housing, nutrition, care, and welfare, and of equal probability of BPV exposure and other risk factors were assigned to each sarcoid-positive horse as controls. Altogether, 13 horses with clinically manifest sarcoids (positive group) and 30 control horses (negative group) were analyzed.

### BPV1/BPV2 diagnostics

The presence of BPV1/BPV2 was analyzed using swabs collected from sarcoids of all positive positive horses (n = 13) and from 25 sarcoid negative horses by gently rubbing the lesion with a sterile swab for 30 seconds. For the control group of horses, a combined swab was taken from all predilection sites for equine sarcoids. The swabs were then frozen at − 80°C prior to the extraction of total DNA using the Nucleospin Blood kit (MACHEREY-NAGEL, Dueren, Germany). The reaction mixture for qPCR was prepared according to the manufacturer’s instructions using nucleotide primers “BPV qPCR SG forward” 3’GGAATGAACATTTCCGCTTTAC5’ and “BPV qPCR SG reverse” 3’GCAGCAACTAGTCCCAAGAA5’ (targeting the E5 protein sequence) and employing Luna Universal Mastermix reagents (New England Biolabs, Ipswich, USA). The qPCR reaction proceeded under the following conditions: Initial denaturation at 95 °C for 60 s, followed by 45 cycles of denaturation at 95 °C for 15 s and extension at 60 °C for 30 s (with plate read), followed by a melt curve from 60 to 95 °C. Samples were considered positive if the C_t_ value was below 33 cycles. The discriminatory limit (cutoff value) at the 33rd cycle was established based on the analysis of serially diluted plasmid DNA containing the BPV E5 oncoprotein gene sequence at a known concentration. All thirteen horses diagnosed with sarcoids were positive for BPV1/2; all 25 sarcoid negative horses examined were negative for BPV1/2.

### Blood samples and DNA extraction

Peripheral blood samples were collected on the occasion of standard routine diagnostic procedures performed on a regular basis in the studs followed. DNA was extracted using the NucleoSpin Blood kit (Macherey–Nagel, Düren, Germany) according to the manufacturer’s instructions.

### Microsatellite markers genotyping

Fourteen microsatellites located in the MHC class I, II and III subregions, and eleven microsatellites in the KLRA subregion of the NKC were analyzed as described in Horecky et al. (2018) (Table 1).

**Table 1:** Microsatellites and candidate genes used in the study and their genomic localization

Locus	Genomic region (KLRA)	Chromosome position	References	Locus	Genomic region (MHC)	Chromosome position		References	Locus (parentage panel)	Genomic region
*ABGe3660*	ECA6:KLRA	42 216 353	Mittmann et al. (2010)	*TKY3324*	ECA20:MHC II	33 142 165		Brinkmeyer-Langford et al. (2013)	*VHL20*	ECA30
*TKY1745*	ECA6:CLEC	36 201 468	Tozaki et al. (2007)	*COR112*	ECA20:MHC II	33 282 616		Tseng et al. (2010)	*HTG4*	ECA 9
*CZM005*	ECA6:KLRA	37 761 959	Horecky et al. (2018)	*COR114*	ECA20:MHC II	33 516 438		Tseng et al. (2010)	*AHT4*	ECA 24
*CZM006*	ECA6:KLRA	38 182 721	Horecky et al. (2018)	*UM011*	ECA20:MHC II	33 510 118		Tseng et al. (2010)	*HMS7*	ECA 1
*CZM007*	ECA6:KLRA	38 418 893	Horecky et al. (2018)	*UMN-JH34 - 2*	ECA20:MHC I	29 232 117		Tseng et al. (2010)	*HMS6*	ECA 4
*CZM008*	ECA6:KLRA	38 487 953	Horecky et al. (2018)	*305 − 93*	ECA20:MHC I	29 289 063		Brinkmeyer-Langford et al. (2013)	*ASB23*	ECA 3
*CZM009*	ECA6:KLRA	39 965 005	Horecky et al. (2018)	*CZM001*	ECA20:MHC I	29 980 161		Horecky et al. (2018)	*ASB2*	ECA 15
*CZM010*	ECA6:KLRA	40 098 138	Horecky et al. (2018)	*CZM002*	ECA20:MHC I	30 013 021		Horecky et al. (2018)	*HTG10*	ECA 21
*CZM011*	ECA6:KLRA	40 538 335	Horecky et al. (2018)	*CZM003*	ECA20:MHC I	31 094 548		Horecky et al. (2018)	*HMS3*	ECA 9
*CZM012*	ECA6:KLRA	41 145 725	Horecky et al. (2018)	*CZM004*	ECA20:MHC II	32 689 856		Horecky et al. (2018)	*HMS2*	ECA 10
*CZM013*	ECA6:KLRA	41 794 947	Horecky et al. (2018)	*ABGe17402*	ECA20:MHC I		30 501 074	Mittmann et al. (2010)	*HTG7*	ECA 4
				*TKY2933*	ECA20:MHC I		30 600 154	Tozaki et al. (2007)	*ASB17*	ECA 5
				*ABGe9019*	ECA20:MHC III		31 385 172	Brinkmeyer-Langford et al. (2013)	*LEX3*	ECA X
				*HMS082*	ECA20:MHC III		31 475 089	Brinkmeyer-Langford et al. (2013)	*HMS1*	ECA 15
				*DRA*	ECA20:MHC II		32 690 816	Plasil et al. 2023	*CA425*	ECA 28
				*DQA1*	ECA20:MHC II		32 789 723	Plasil et al. 2023		

In addition, fifteen microsatellite loci used for parentage testing in horses were analyzed as controls: *AHT4*,* ASB2*,* ASB17*,* ASB23*,* CA425*,* HMS1*,* HMS2*,* HMS3*,* HMS6*,* HMS7*,* HTG4*,* HTG7*,* HTG10*,* LEX3*, and *VHL20* (Table 1). The positions of the markers on the chromosomes are based on the positions stated in the references and correspond to the EquCab2 assembly. Multiplex PCR was performed using the commercially available Equine Genotypes Panel 1.1 (Thermo Fisher Scientific Inc., Waltham, USA) according to the manufacturer’s instructions. Each 0.5 µl of the 30x diluted PCR product and 0.1 µl of the GeneScan™ 600 LIZ^®^ Size Standard dye (Life Technologies) were loaded in 11.3 µl of Hi-Di™ Formamide (Life Technologies, Carlsbad, CA, USA). The samples were then denatured at 95 °C for 3 min and cooled down. The genotype scoring was performed on an ABI PRISM 3500 Genetic Analyzer (Applied Biosystems) with five-color detection by fluorescent fragment analysis and evaluated using the 3500 Data Collection software 3.3 and GeneMapper 6.0. Samples of known genotypes were used as references and positive controls to standardize the allele sizes.

### Candidate MHC class II gene genotyping

Primers amplifying exon 2 of the *DRA* and *DQA1* genes and PCR protocols were the same as in our previous study (Plasil et al. 2023). Amplicons from each horse were individually indexed and sequenced on Illumina MiSeq, using the Nextera XT Library Prep Kit (Illumina, USA). Sequencing provided paired-end reads up to 250 bp long. Each base position was covered by at least 30 reads; in most cases, the coverage was higher (> 200×). Novel *DQA1* alleles were submitted to NCBI under accession numbers PQ336833-PQ336842.

### Long-read sequencing of a candidate region

To further investigate the most significant association with the marker *COR112*, a 5,4 kb region surrounding this locus was amplified. Primer3web (Untergasser et al. 2012) and Primer-BLAST were used for primer design based on the horse reference genome sequence. Primer sequences and additional information can be found in Online Resource 1. Amplification of the 5407 bp long product was carried out using Expand Long Range dNTPack (ROCHE Diagnostics, Mannheim, Germany) according to the manufacturer’s recommendations and in a 12.5 µl total volume. PCR amplicons were sequenced on MinION, using Ligation sequencing kit (Oxford Nanopore, Oxford, United Kingdom).

### NGS and sequence analysis

Standard Illumina MiSeq and MinION sequencing were carried out as described previously (Plasil et al. 2023). *DRA* and *DQA1* alleles, variable positions, and haplotypes in the 5,4 kb *COR112* region were manually reconstructed from the alignment data using IGV 2.8.2. The nomenclature rules established in the IPD/MHC database (Maccari et al. 2017) were followed for the identification of *DRA* and *DQA1* alleles. Based on the EquCab3 (GCF_002863925.1) and TB-T2 T (GCF_041296265.1) assemblies, both the NCBI database (Sayers et al. 2021) and the RNAcentral database (RNAcentral Consortium 2021) were searched to identify potential regulatory RNAs in the proximity of the *COR112* microsatellite.

### Statistical analyses

Haplotypes were inferred using the ELB algorithm of the Arlequin 3.5 software (Excoffier and Lischer 2010). Differences between the groups (mean age) were analyzed by a standard Mann-Whitney-Wilcoxon test using an online calculator (https://astatsa.com/WilcoxonTest/).

The standard two-sided Fisher’s exact and Chi-square tests were used to assess associations between individual alleles and/or genotypes and the presence of clinical sarcoid. Bonferroni corrections for multiple comparisons were made. Only associations with Bonferroni-corrected P-values < 0.05 were considered significant. Additionally, associations were analyzed for two types of haplotypes: shorter haplotypes consisting of two adjacent markers from the same subregion, and longer haplotypes composed of all analyzed microsatellites from the same subregion. The Bonferroni corrections for the shorter haplotypes were based on the number of alleles for both included markers with a frequency greater than 0.10, plus one containing all remaining low-frequency alleles. The Bonferroni correction for the longer haplotypes was based on the number of analyzed haplotypes with a frequency surpassing 0.10 plus one containing all other low-frequency haplotypes.

To identify possible interactions between the MHC and KLRA regions, the combinations of microsatellite MHC and KLRA alleles and their associations with sarcoids were analyzed according to de Wit et al. (2011), as described previously (Stejskalova et al. 2019). Bonferroni corrections were not used in this situation since they are considered too conservative for association tests of genetic polymorphisms located on different chromosomes (de Wit et al. 2011; Nyholt 2004). Only associations of compound genotypes with P-values at least ten times lower than the P-values calculated for each individual marker were considered informative.

## Results

Statistically significant results showed associations between the presence of sarcoid with different microsatellite markers of the MHC class II genomic sub-region (P_corr_< 0.05, P_corr_< 0.01) and with compound MHC class I_KLRA microsatellite genotypes (*P* < 0.01). No significant associations were found for MHC class I, class III and KLRA microsatellite loci, for MHC class II expressed loci *DRA* and *DQA1*, or for non-MHC microsatellite markers used for parentage testing.

### Results of association analyses by sub-regions

#### MHC class II

Significant associations (P_corr_< 0.05, P_corr_< 0.01) were found between the presence of sarcoid and the MHC class II microsatellites *CZM004*,* TKY3324*, and *COR112* (Tables 2 and 3).

**Table 2:** MHC microsatellites associations with sarcoid in 13 sarcoid positive and 30 sarcoid negative Arabian horses

Msat^1^	F_ST_	*P*	No. of alleles^2^	Alleles associated	*P*_uncorr_	*P*_corr_	Effect
MHC II	0.039	0.005	-	-	-	-	-
*CZM004*	0.142	0.002	3	118, 122	**0.005**, 0.016*	**0.020**, 0.048*	**R**,** S**^3^
*TKY3324*	0.036	0.039	3	246	**0.006**	**0.018**	**R**
*COR112*	0.120	0.004	4	244, 252, 254	**0.006**, 0.002* 0.024	**0.024**, 0.008* 0.096	**R**,** R** S
*UM11*	0.220	0.001	4	164, 168, 180	**0.019**, 0.014* 0.035	0.076, 0.056* 0.140	R, R R
*COR114*	0.076	0.030	6	233	**0.035**	0.210	R
MHCI	0.045	0.060	-	-	-	-	
*CZM001*	0.090	0.021	5	176	**0.025**	0.130	R
*TKY2933*	0.140	0.022	5	217	**0.024**	0.118	R
*305 − 93*	0.074	0.017	4	126	**0.014**	0.056	R

^1^ microsatellite; ^2^ number of alleles; ^3^ resistance, susceptibility

* Two P-values within the P_uncorr_ and P_corr_ columns referr to associations of alleles identified in the previous column

**Table 3:** Differences in frequencies of significantly associated MHC class II microsatellite alleles between sarcoid-negative and sarcoid-positive horses

Microsatellite locus	Allele	Effect		Sarcoid-negative horses (*N* = 30)		Sarcoid-positive horses (*N* = 13)	
			*P*_corr_	*N* ^1^	Allele frequency	*N*	Allele frequency
*CZM004*	118	R^2^	0.020	27	0.767	7	0.385
	122	S^3^	0.048	7	0.133	8	0.423
*TKY3324*	246	R	0.018	30	0.850	9	0.615
*COR112*	244	R	0.024	25	0.433	5	0.231
	252	R	0.008	20	0.333	2	0.115

^1^ number of animals; ^2^ resistance; ^3^ susceptibility

None of the associated MHC class II microsatellite alleles were private, i.e. they did not occur exclusively in only one of the analyzed groups. All but one of the MHC class II microsatellite associated alleles were associated with resistance to the disease. The exception was *CZM004/122.*

The significant associations of individual MHC class II microsatellites were confirmed and extended by the analysis of haplotypes containing a particular associated marker with a neighboring microsatellite (Table 4).

**Table 4:** Associations of haplotypes containing the associated marker with a neighboring microsatellite in 13 sarcoid positive and 30 sarcoid negative Arabian horses

MHC I	Marker combinations	Sarcoid		Effect	*P*_uncorr_	*P*_corr_	Alleles
		Positive *N*^1^	Negative *N*				
Hap A	*CZM001_CZM002*	0	9	-	**0.039**	0.780	176_246
Hap B	*TKY2933_ABGe17402*	0	10	-	**0.020**	0.490	217_211
Hap C	*305 − 93_UMN-JH*	0	9	-	**0.039**	0.626	126_208
Hap D	*305 − 93_CZM001*	1	15	-	**0.014**	0.286	126_176

MHC II	Marker combinations	Positive *N*	Negative *N*	Effect	*P*_uncorr_	*P*_corr_	Alleles
Hap E	*CZM004_TKY3324*	6	27	R^2^	**0.004**	**0.035**	118_246
Hap F	*COR114_UM011*	5	22	-	**0.043**	1.030	233_180
Hap G	*TKY3324_COR112*	4	25	R	**0.001**	**0.021**	246_244
Hap H	*COR112_UM011*	4	24	-	**0.004**	0.082	244_168

^1^ number of animals; ^2^ resistance

After correction, the combinations of markers *CZM004_TKY3324* and *TKY3324_COR112* remained associated (P_corr_< 0.05). Further, haplotypes of the entire MHC class II region (Online Resource 1) were analyzed. A total of 23 haplotypes were identified, with 4 occurring at a frequency higher than 0.10. Among these, the haplotype *CZM004/118_TKY3324/246_COR112/244_UM11/168_COR114/237* was associated with the presence of sarcoids before correction (P_uncorr_< 0.05), but was no longer significant after correction.

As for the expressed MHC class II genes *DRA* and *DQA1*, no significant association of their alleles and/or homozygous genotypes with equine sarcoid were observed at P_corr_< 0.05. The homozygous Eqca-DRA* 001:01/Eqca-DRA* 001:01 genotype was found only in the group of positive horses. However, its association with sarcoid was significant only at P_uncorr_< 0.05 (Table 5).

**Table 5:** Distribution of the MHC class II allele DRA* 001:01 in Arabian horses with and without sarcoid

Alleles and genotypes	Associations			Sarcoid			
				Positive (*N* = 13)		Negative (*N* = 30)	
	P_uncorr_	P_corr_	Effect	N^1^	Frequency	N	Frequency
Allele Eqca-DRA* 001:01	0.057	0.228	S^2^	9	0.433	13	0.217
Genotype DRA* 001:01/Eqca-DRA* 001:01	**0.024**	0.192	S	2	0.153	0	0.000

^1^ number of animals; ^2^ susceptibility

Out of the nineteen SNPs (MAF > 0.10) identified in the 5 kb region surrounding the *COR112* microsatellite in Arabian horses, seven SNPs, occurring in five haplotypes, were associated with sarcoid at P_corr_ < 0.01 (Online Resource 1). The associated SNPs are located at the following positions of the sequence of chromosome 20 in the TB-T2T genome: 36,700,259, 36,700,338, 36,700,406, 36,700,410, 36,701,386, 36,701,482, and 36,701,710. In the negative horses, one SNP haplotype completely predominated (G_T_A_A_G_A_C at the aforementioned positions), occurring in at least one copy in each negative animal. When including the *COR112* microsatellite, the number of haplotypes (7SNP plus *COR112*) increased to 16. In the negative horses, two of these combined haplotypes predominated. They include the same SNPs alleles (G_T_A_A_G_A_C) but differ in the *COR112* allele, which is either 244 or 252. Every negative animal except one had at least one of these predominant haplotypes. In positive animals, these two haplotypes were present in 40% of the horses. Both of these haplotypes were associated (Table 6) with sarcoid (P_corr_< 0.05, P_corr_< 0.01).

**Table 6:** Associations of SNP haplotypes surrounding *COR112* microsatellite in 13 sarcoid positive and 30 sarcoid negative horses

Haplotype	Positions in the sequence of chromosome 20 in the TB-T2 T 36700259_ 36700338_ 36700406_ 36700410_ 36701386_ 36701482_ 36701557 - 602_ 36,701,710	Effect	Animals		*P*_uncorr_	*P*_corr_
			Sarcoid positive *N* ^1^	Sarcoid negative *N*		
1	*G_T_A_A_G_A_244_C*	R^2^	3	22	**0.002**	**0.007**
2	*G_T_A_A_G_A_252_C*	R	2	17	**0.012**	**0.036**

^1^ number of animals; ^2^ resistance

### Results of association analyses by sub-regions

#### MHC class I

No significant associations of MHC class I microsatellite markers with the presence of sarcoid were found at P_corr_< 0.05 (Table 2).

Associations of two neighboring microsatellites with sarcoid were not significant after correction (Table 4). Out of twenty-four haplotypes identified across the entire MHC class I region (Online Resource 1) four occurred in a frequency higher than 0.10. Among these, the haplotype *UMN-JH34 - 2/208_305 − 93/126_CZM001/176_CZM002/246_ABGe17402/211_ TKY2933/217* was associated with sarcoid before correction (P_uncorr_< 0.05), but not after correction.

### Results of association analyses by sub-regions

#### MHC class III

The MHC class III microsatellite markers were not associated with the presence of sarcoid at P_uncorr_< 0.05 (Online Resource 1).

Associations of sarcoid with pairs of neighboring microsatellites were not observed. Out of ten haplotypes identified across the entire MHC class III region (Online Resource 1), six occurred in a frequency higher than 0.10. None of these haplotypes were associated with sarcoid at P_uncorr_< 0.05.

### Results of association analyses by sub-regions

#### KLRA

Associations with the presence of sarcoid were not detected for any *KLRA* microsatellite markers (Online Resource 1) at P_uncorr_< 0.05.

Seventeen haplotypes of the *KLRA* region were identified; the frequency of six of them surpassed 0.10. None of them were associated with sarcoid at P_uncorr_< 0.05.

### Results of association analyses by sub-regions

#### MHC_KLRA allele combinations

MHC class I_KLRA microsatellite allele combinations were associated with sarcoid at *P* < 0.01 (Table 7).

**Table 7:** Associations of MHC class I_KLRA microsatellites combinations with sarcoid in 43 Arabian horses

Individual MHCI marker allele	*P*_uncorr_	*P*_corr_	MHCI_KLRA alleles combination	*P*	*R*/S
*305 − 93* 126	**0.014**	0.056	*305 − 93_CZM005* 126_269	**0.003**	R^1^
*TKY2933* 217	**0.024**	0.118	*305 − 93_CZM11* 126_151	**0.004**	R
			*TKY2933_CZM005* 217_269	**0.004**	R

^1^ resistance

Associations of MHC class II_KLRA combinations were not informative, as they did not meet the criterion of a tenfold reduction in P-value relative to the individual MHC class II markers (Online Resource 1).

### Non-MHC microsatellite markers

No associations significant at P_corr_< 0.05 were found for the microsatellite markers used for parentage testing (Online Resource 1).

## Discussion

Arabian horses are among the breeds at high risk of developing sarcoids (Mohammed et al. 1992). As such, they represent a suitable model for studying genes underlying inter-breed and individual variation in susceptibility to the disease. In this study, we analyzed associations of selected candidate markers in this breed.

So far, multiple studies have reported on inter-breed and within-breed variation in the prevalence of sarcoid. Genetic analyses of this common and important skin disease have provided different, but not always comparable, and often even conflicting results (reviewed by Ogłuszka et al. 2021). There are four likely contributors to this situation: the heterogeneity of clinical disease in terms of its manifestation and causes; the use of different breeds, reflecting different populations with different epidemiological situations; the use of different methods for association analyses as well as the year of the study. Consequently, different phenotypes and sometimes different genes were studied. As the etiology and pathogenesis of sarcoids have not yet been fully clarified, most candidate gene association studies have focused on immunity-related genes, based on the fact that immune functions are clearly altered in affected horses. The earliest studies identified the MHC region as a strong candidate region underlying differences in the presence of sarcoids (Broström 1995; Broström et al. 1988). Since then, the effects of the MHC region have been repeatedly confirmed by candidate genes studies as well as by genome-wide approaches. GWAS has also confirmed that multiple non-MHC genes are involved in the mechanisms of susceptibility to sarcoid (Staiger et al. 2016). Most but not all MHC studies have found the most significant associations with the MHC class II sub-region, including across different breeds. In this study, we used microsatellite markers characterizing the diversity of all three MHC subregions (Carr et al. 2001; Martens et al. 2001) to distinguish between their effects on the manifestation of sarcoid. The results showed significant associations of sarcoid with several markers of the MHC class II region. Less significant and non-significant associations were observed for the class I and class III sub-regions, respectively. These data may be interpreted as resulting from the strong effect of the MHC class II sub-region and for the MHC class I as a result of linkage disequilibrium (LD) with MHC class II (Table 2). However, there is also the possibility that they represent a primary but weak effect of MHC class I genes in the cohort studied. Associations with MHC class I serological haplotypes reported in the past (Broström et al. 1988; Lazary et al. 1994) may be explained in the same way. The data presented here found that although associations with individual MHC class I loci were always non-significant after corrections, their combinations with equally non-significant KLRA markers led to a decrease of the P value below 0.01.

There are several limitations of the findings that must be considered in their interpretation. Sarcoids were diagnosed and classified through a standard clinical examination. Aiming to study clearly distinct phenotypes, we did not include information regarding the heterogeneity of their clinical manifestation and studied associations between the presence/absence of clinical sarcoid in three herds with a permanent presence of both phenotypes. Due to ethical and logistical limitations of the project, the presence of BPV1/2 in biopsies of pathologically altered tissues could not be examined. The presence of BPV1/2 was detected from swabs of all sarcoid positive and was not found in swabs from negative horses. These results are consistent with previous reports. It has been shown that viral DNA can be found on sarcoids as well as on normal skin of horses affected with sarcoids, and to a lesser extent on the normal skin of healthy horses (Bogaert et al. 2005).

The average age of sarcoid-positive horses in this study was 10 years (4–19 yrs), while the average age of negative horses was 13 years (5–27 yrs). The age difference between the two groups was significant at *P* < 0.004, which means that negative horses were exposed to the complex pathogenic risk of sarcoid longer than the positive ones, yet they did not develop clinical disease. Considering that sarcoid is mostly a disease of young adult animals, commonly seen between 3 and 6 years of age (Broström 1995; Marti et al. 1993) and in some studies also commonly diagnosed at 7 to 8 years (Knowles et al. 2016; Wobeser et al. 2010), negative adult horses may be considered to be more resistant.

The sample size may also have influenced the results of association analyses. The number of horses reflects the availability of clinical cases in the selected studs within the timeframe of the project. To reduce the potential effects of this factor, we have used a two-fold number of negative control horses and applied overconservative (Bonferroni) corrections with only results at P_corr_< 0.05 interpreted as significant findings. No microsatellite loci from the parentage kit were associated with the presence of sarcoid. It is possible that these procedures increased the risk of false negative results, e.g. for MHC class I and KLRA data, but they significantly reduced the risk of false positive results.

Due to the limited availability of clinical cases, some of the horses, positive as well as negative, were related. However, the frequencies of associated alleles of MHC microsatellite loci and of *DRA* and *DQA1* genes in related horses were not different from those in unrelated horses (*P* > 0.05, Chi^2^ test), with the exception of the *COR112/252* microsatellite allele. This allele, associated with resistance to sarcoid, was absent in related negative horses (*n* = 8). These results suggest that the accumulation of associated alleles is not primarily a reflection of the relatedness of the horses under study but rather of the occurrence of sarcoids.

Respecting the above limitations, the significant associations of the analyzed markers with the presence of sarcoid can be interpreted in terms of their biological plausibility.

Association studies of papilloma virus-associated tumors in humans identified the MHC class II region as a factor contributing to the susceptibility to these diseases (Bao et al. 2018; Chuang et al. 2012; Espinoza et al. 2021). MHC class II markers have been repeatedly associated with equine sarcoid (Hörnaeus 2013; Lazary et al. 1994; Ogłuszka et al. 2021; Staiger et al. 2016). Despite the heterogeneity of these studies, our data are consistent with their conclusions. MHC class II molecules present peptides derived from extracellular proteins to T lymphocytes. According to Chambers et al. (2003), specific MHC class II alleles may be associated with impaired immune responses to BPV and/or other sarcoid tumor-associated antigens. However, no definitive conclusions have been reached regarding specific sub-regions and/or loci in the horse genome which could be interpreted as causative variants explaining known associations in the context of gene function. We studied the variability of the MHC class II region by using microsatellite and expressed gene markers located in different segments of the class II region. Most of the associated MHC class II microsatellite alleles were associated with resistance to the disease, with only one associated with susceptibility. This might be due to the higher number of negative horses in the cohort. With these data, we can confirm that in the MHC class II region, there are sequences contributing to resistance and susceptibility to the development of sarcoid. In this particular cohort, the associated region was delimited by microsatellites *CZM004– COR112* (36.11–36.71 Mb of chromosome 20 in TB-T2T genome), covering approximately a 0.6 Mb of chromosome 20, and containing expressed genes (*DRA*,* DRB*,* DQA*,* DQB*), pseudogenes, microsatellites, and RNA non coding sequences (lncRNA, microRNA).

Candidate MHC class II genes *DQA*,* DQB*,* DRA*, and *DRB* encode antigen-presenting molecules expressed on the surface of different types of immune cells. Their antigen-binding site is encoded by exon 2 of their respective genes. The most commonly analyzed equine class II genes are *DRA* and *DQA1* (Janova et al. 2009; Kamath and Getz 2011), which are located in close proximity (approx. 10 kb) to the associated microsatellite *CZM004*. All associations of these genes were non-significant after corrections. It is possible that this is a false negative result, but it is also possible that it is not the exon 2 nucleotide sequence which is involved in the anti-sarcoid immune mechanisms. Staiger et al. (2016) described a potential regulatory polymorphism in an intron of the *DQA1* gene in horses. This part of the *DQA1* sequence was not analyzed in this study.

A detailed analysis of the 5.4 kb segment containing the strongly sarcoid-associated microsatellite *COR112* sequence identified seven SNPs and their haplotypes associated with the presence of sarcoid. According to the transcriptome data (BioProject PRJNA1135235, https://www.ncbi.nlm.nih.gov/bioproject/PRJNA1135235), the *COR112* region (Eqca chromosome 20: 36701412–36701695) is located close to two functional genes, *DRB2* (76.5 kb) and *DRB3* (75 kb). It is not clear whether the *COR112*-sarcoid associations are due to the effects of closely linked genes or to non-coding and/or regulatory sequences, such as miRNA or lncRNA.

As for protein-coding genes, an association of the human *HLA-DRB1* gene with papillomavirus-induced tumors was reported (Leo et al. 2017), but no information on expressed *Eqca* genes is available. On the other hand, sequences related to several miRNA precursors are located approximately 6 kb upstream and 1 kb downstream of *COR112*, and an annotated lncRNA (Gene ID: *102150801 lncRNA* - NCBI) is located about 40 kb downstream (The RNAcentral database, Online Resource 1). We can only speculate about their potential role in the regulation of sarcoid development. Semik-Gurgul et al. (2023) described differentially expressed lncRNAs in equine sarcoid on chromosome 20, with the nearest lncRNA (*ENSECAG00000048295*) located approximately 0.6 Mb from the *COR112* microsatellite sequence. However, the most important differentially expressed lncRNAs identified in that study are located in the 7.23–7.31 Mb region of chromosome 20, outside the MHC region.

None of the MHC class I markers were associated with the presence of sarcoid. However, there is evidence that the MHC class I sub-region is associated with equine sarcoids (Broström 1995; Broström et al. 1988; Meredith et al. 1986). Previous studies have also confirmed associations of this sub-region with human papillomavirus infections and tumors (Bao et al. 2018; Espinoza et al. 2021; Leo et al. 2017; Metcalfe et al. 2013). According to Bao et al. (2018), genetic factors located in both MHC class I and class II sub-regions play important roles in the pathogenesis of human squamous cell carcinoma. The negative results of this study may be due to the rather small number of positive cases available and/or to the relatively weaker effects of the MHC class I sub-region compared to class II.

Nevertheless, since MHC class I molecules play several roles in anti-virus and anti-tumor immunity, including presenting viral and tumor-associated antigens to T lymphocytes that elicit immune responses to infected and/or transformed cells, they are likely to impact the pathogenesis of equine sarcoids. E5, the main oncoprotein of BPV1 in equine sarcoids, was shown to reduce the expression of MHC class I molecules on the surface of tumor cells (Marchetti et al. 2009). The loss of antigen presentation through MHC class I molecules is one of the mechanisms tumors immunoevasion (Dhatchinamoorthy et al. 2021), resulting in poor immune clearance of tumor cells, lack of spontaneous regression, and tumor recurrence, which is supposed to occur in equine sarcoids as well (Ogłuszka et al. 2021).

In humans, specific combinations of MHC class I and NK cell receptor alleles are associated with several diseases, including virus-derived tumors (Carrington et al. 2005; Pollock et al. 2022). Some combinations may be of adaptive value; MHC class I and NK receptor genes thus co-evolve, even though they are located on different chromosomes (Guethlein et al. 2015). Based on our previous observations of non-random associations between MHC and NKR polymorphic markers in horse populations (Bubenikova et al. 2024), we tested the hypothesis that compound MHC class I_KLRA microsatellite marker genotypes are associated with equine sarcoid. When tested independently, no association at P_corr_< 0.05 was found for either KLRA or MHC class I markers. However, several MHC class I_KLRA microsatellite allele combinations were associated with the disease. Compound MHC class I_KLRA genotypes reached statistical significance at *P* < 0.01 (Table 7), higher by one order of magnitude compared to the P-values observed for the two loci tested independently. Associations of MHC class II_KLRA microsatellite combinations had similar levels of significance as individual MHC class II microsatellites, suggesting that the significance is driven by the MHC class II microsatellites alone, as the significance does not increase in genotype combinations as would be expected with synergistic effects (de Wit et al. 2011).

Sarcoid is a virus-induced tumor resulting from the interaction between viral infection, the environment, and the host genome. According to Jindra et al. (2023), the rejection of BPV-associated lesions is largely mediated by the cellular immune response. It seems that the sarcoid progression in horses is associated with the disruption of both the innate and adaptive immune responses; a key process in the initiation of the immune response is antigen presentation by MHC molecules (Yuan et al. 2008). In this context, the data presented here may be interpreted as a result of effects of both specific, MHC class II mediated immunity and at the same time of innate immunity involving KLRA molecules on infected and/or transformed cells. This interpretation is supported by the results of the currently available GWAS and transcriptome studies (Semik et al. 2017; Yuan et al. 2008). The data also can help to interpret the sometimes-conflicting results of association studies observing both MHC class I and II associations with equine sarcoid and may contribute to a better understanding of immune responses to sarcoid. In humans, the HLA class I, HLA class II and NKR genomic regions are associated with papillomavirus-derived tumors (Bao et al. 2018; Tembhurne et al. 2023). Deficiencies in the NK cell response to human papillomavirus infection have also been reported, but the underlying mechanism has not yet been defined (O’Brien and Saveria Campo 2002).

The results report on associations between NKR and MHC microsatellite markers in the Arabian breed. However, no associations with the same markers were observed in Thoroughbreds and Warmbloods (data not shown), due probably to haplotype variability between breeds. Further detailed analyses are needed to explain the differences observed.

## Conclusion

Associations of MHC class II and MHC class I_KLRA polymorphic markers with the presence of sarcoid were observed. Despite some limitations of the study, and considering the risk of false negative results, the significant results are nevertheless biologically plausible and indicate a role of MHC class I, class II and KLRA molecules in adaptive as well in innate immune responses to equine sarcoid. The data point to an as yet unexplored hypothesis on the possible roles of NK cells on the pathogenesis of equine sarcoid. The data presented here are limited to Arabian horses; based on the current literature, large interbreed differences may be expected. The focus of this study was primarily the MHC genomic region, and secondarily the NKC region. However, GWAS and transcriptome data clearly show that the pathogenesis of equine sarcoid is complex and further research is needed to cope with the complexity of the disease as well as with the complexity of the associated immune responses and their underlying genes and genomic regions.

## Electronic supplementary material

Below is the link to the electronic supplementary material.

Supplementary Material 1 Online Resource 1 (pdf)– *COR112* region primers, haplotype, non-MHC msat and SNP associations with sarcoid.

## Data Availability

All data supporting the findings of this study are available within the paper and its Supplementary Information (Online resource 1); original references describing the microsatellites and MHC genes used in this study are in https://onlinelibrary.wiley.com/doi/full/10.1111/tan.13211 and https://onlinelibrary.wiley.com/doi/10.1111/tan.15078, respectively. Further data supporting the findings of this study are available from the corresponding author upon reasonable request.
