# Supplementary material for: Genetic susceptibility to sarcoid in Arabian horses: associations with MHC class II and compound MHC class I/KLRA genotypes
Source: Vet Res Commun. 2025 May 1;49(3):184. doi: 10.1007/s11259-025-10748-2 (PMC12045807; doi:10.1007/s11259-025-10748-2)
Supplement: Supplementary file 1 — Supplementary Material 1 Online Resource 1 (pdf)– COR112 region primers, haplotype, non-MHC msat and SNP associations with sarcoid. [file 11259_2025_10748_MOESM1_ESM.pdf]

Genetic susceptibility to sarcoid in Arabian horses: associations with MHC class II and compound MHC class I/KLRA genotypes

Veterinary Research and Communications

Leona Vychodilova[1], Martin Plasil[2], Jan Futas[1,2], Andrea Kopecka[1], Dobromila Molinkova[1], Tamara Wijacki[3], Petr Jahn[1], Ales Knoll[3], Petr Horin[1,2 *]

[1]Faculty of Veterinary Medicine, University of Veterinary Sciences, Brno, Czech Republic

[2]Ceitec Vetuni, University of Veterinary Sciences, Brno, Czech Republic

[3]Faculty of Agronomy, Mendel University, Brno, Czech Republic

Corresponding author email: horin@med.muni.cz

Table S1 Primers

Primers	Sequence	PCR product length	Ta (°C)	Position NC_009163 CHr 20 EquCab 3.0	Position Chr 20 TB-T2T
COR112	5´ccccaagcagaccaaacttc3´/<br>5´agggtctgagagggaaaacg3´	5406	62	34186982bp<br>34192388bp	36696389bp<br>36701793bp

Table S2 Haplotypes across the entire MHC or NKC region

UMN-JH34-2 305-93 CZM001 CZM002 ABGe17402 TKY2933			Sarcoid			
MHCI **Most common haplotypes**	**Absolute frequency**	**Alleles**	**positive**	**negative**	P_{uncorr}	P_{corr}
≥10%						
1	10	204 128 174 248 205 209	4	6	NS	NS
2	14	214 128 180 234 209 221	4	10	NS	NS
4	17	204 128 176 254 219 217	4	13	NS	NS
6	8	208 126 176 246 211 217	0	8	**0.039**	0.195
Number of different haplotypes	24					

CZM004 TKY3324 COR112 UM11 COR114			Sarcoid			
MHCII **Most common haplotypes**	**Absolute frequency**	**Alleles**	**positive**	**negative**	P_{uncorr}	P_{corr}
≥10%						
1	22	118 246 252 180 233	4	18	NS	NS
2	10	122 256 246 168 237	4	6	NS	NS
4	15	118 246 244 168 237	1	14	**0.014**	0.055
9	6	118 246 244 168 231	1	5	NS	NS
Number of different haplotypes	23					

CZM003 ABGe9019 HMS082			Sarcoid			
MHCIII **Most common haplotypes**	**Absolute frequency**	**Alleles**	**positive**	**negative**	P_{uncorr}	P_{corr}
≥10%						
1	10	285 304 207	4	6	NS	NS
2	20	285 310 205	7	13	NS	NS
4	22	285 292 205	7	15	NS	NS
5	12	285 304 209	2	10	NS	NS

6	7	282 298 207	1	6	NS	NS
7	6	282 308 209	1	5	NS	NS
The number of different haplotypes	10					

**CZM005 CZM006 CZM007 CZM008** *NKC* Most common haplotypes	**Absolute frequency**	**Alleles**	**Sarcoid** positive	negative	P_{uncorr}	P_{corr}
≥10%						
1	18	271 222 242 267	3	15	NS	NS
2	11	269 220 242 261	1	10	NS	NS
3	16	269 218 242 265	4	12	NS	NS
6	9	269 222 242 267	4	5	NS	NS
4	6	273 218 242 267	2	4	NS	NS
5	6	269 222 242 265	0	6	NS	NS
The number of different haplotypes	17					

Table S3 Associations of individual SNPs within the *COR112* region

**SNP position chrom. 20 in the**		**Allele**	**Effect**	P_{uncorr}	P_{corr}
**EquCab3**	**TB-T2T**				
34190845	36700259	G	R	$4*10^{-5}$	$9.75*10^{-5}$
34190924	36700338	T	R	0.002	0.003
34190992	36700406	A	R	0.004	0.008
34190996	36700410	A	R	0.004	0.008
34191971	36701386	G	R	0.004	0.008
34192067	36701482	A	R	0.0007	0.002
34192285	36701710	C	R	0.0007	0.002

R resistance

Table S4 Associations of MHC class III microsatellites

Microsatellite	FST	CHi2 P
MHCIII	0.000	NS
HMS 082	0.000	NS
ABGe 9019	0.000	NS

NS – non significant

Table S5 NKC *a*ssociation analysis

Microsatellite	subregion	FST	CHi2 P
CZM011	KLRA	0.004	NS
CZM006	KLRA	0.015	NS
CZM005	KLRA	0.002	NS
TKY1745	CLEC	0.000	NS
CZM009	KLRA	0.022	NS
CZM010	KLRA	0.000	NS
CZM008	KLRA	0.000	NS
CZM012	KLRA	0.061	NS
CZM007	KLRA	0.000	NS
CZM013	KLRA	0.017	NS
ABGe3660	KLRA	0.000	NS

NS – non significant

Table S6 Associations of MHC class II/KLRA microsatellites combinations with sarcoid in 43 Arabian horses

**Individual *MHCII* microsatellite**	**P_{uncorr}**	**P_{corr}**	***MHCII/KLRA* allele combinations**	**P**	**R/S**
TKY3324 246	**0.006**	**0.018**	*COR112 /CZM007* 244/242	**0.006**	R
COR112 244/252/254	**0.006/ 0.002/ 0.024**	**0.024/ 0.008** 0.096	*COR112 / CZM005* 244/269	**0.001**	R
			TKY3324 / CZM005 246/269	**0.001**	R
			TKY3324 / CZM007 246/242	**0.001**	R

R resistance

Table S7 Associations between non-MHC microsatellites and presence of sarcoid

**Msat**	**Eca #**	**P_{uncorr}**	**P_{corr}**	**Msat**	**Eca #**	**P_{uncorr}**	**P_{corr}**
VHL20	30	**0.032**	NS	***HMS3***	9	**0.042**	NS
HTG4	9	NS	NS	***HMS2***	10	NS	NS
AHT4	24	NS	NS	***HTG7***	4	NS	NS
HMS7	1	NS	NS	***ASB17***	5	NS	NS
HMS6	4	NS	NS	***LEX3***	X	**0.021**	NS
ASB23	3	**0.022**	NS	***HMS1***	15	**0.011**	NS
ASB2	15	NS	NS	***CA425***	28	NS	NS
HTG 10	21	NS	NS				

Msat microsatellite; NS non-significant

Table S8 Sequence in COR112 region related to miRNA

miRNA	Accesions no	Eqca 20 location
eca-mir-9152	MI0028375	36695841
eca-mir-9013	MI0028175	36695835
eca-mir-9004	MI0028164	36702611
